# Clinical application of ^18^F-fluorodeoxyglucose positron emission tomography/computed tomography radiomics-based machine learning analyses in the field of oncology

**DOI:** 10.1007/s11604-023-01476-1

**Published:** 2023-08-01

**Authors:** Masatoyo Nakajo, Megumi Jinguji, Soichiro Ito, Atushi Tani, Mitsuho Hirahara, Takashi Yoshiura

**Affiliations:** Department of Radiology, Graduate School of Medical and Dental Sciences, Kagoshima University, 8-35-1 Sakuragaoka, Kagoshima, 890-8544 Japan

**Keywords:** ^18^F-FDG, PET/CT, Radiomics, Machine learning, Oncology

## Abstract

Machine learning (ML) analyses using ^18^F-fluorodeoxyglucose (^18^F-FDG) positron emission tomography (PET)/computed tomography (CT) radiomics features have been applied in the field of oncology. The current review aimed to summarize the current clinical articles about ^18^F-FDG PET/CT radiomics-based ML analyses to solve issues in classifying or constructing prediction models for several types of tumors. In these studies, lung and mediastinal tumors were the most commonly evaluated lesions, followed by lymphatic, abdominal, head and neck, breast, gynecological, and other types of tumors. Previous studies have commonly shown that ^18^F-FDG PET radiomics-based ML analysis has good performance in differentiating benign from malignant tumors, predicting tumor characteristics and stage, therapeutic response, and prognosis by examining significant differences in the area under the receiver operating characteristic curves, accuracies, or concordance indices (> 0.70). However, these studies have reported several ML algorithms. Moreover, different ML models have been applied for the same purpose. Thus, various procedures were used in ^18^F-FDG PET/CT radiomics-based ML analysis in oncology, and ^18^F-FDG PET/CT radiomics-based ML models, which are easy and universally applied in clinical practice, would be expected to be established.

## Introduction

Positron emission tomography (PET)/computed tomography (CT) with ^18^F-fluorodeoxyglucose (^18^F-FDG), a glucose analog that reflects metabolic glucose activity, is widely used in oncology [1]. Radiomics refers to different mathematical methods for extracting several quantitative features to obtain useful biological information [2], and radiomics-based ^18^F-FDG PET has also been applied in oncology [3–6].

The development of artificial intelligence (AI) is associated with relevant psychological, ethical, and medicolegal issues, which should be addressed before AI can be completely considered in patient management. However, the ultra-rapid analysis of large datasets is a major strength of AI in healthcare applications. In the field of medical imaging, AI has been significantly beneficial in predicting individual patient outcomes [7, 8]. Machine learning (ML) can resolve complex interactions among numerous variables to construct a prediction model as accurate as possible [9–11]. The flexibility and scalability of ML are superior to those of conventional statistical approaches. Hence, ML is useful in several tasks including diagnosis and classification.

Recently, the ML or deep learning (DL) models using ^18^F-FDG PET/CT radiomic features have been applied to resolve issues in classification (i.e., “benign or malignant tumor,” “primary or metastatic tumor,” “classification of histological subtypes,” and “recurrence or non-recurrence”) or to construct prediction models (i.e., “tumor characteristic,” “tumor stage,” or “survival”) [12]. The current review aimed to summarize the current clinical studies on ^18^F-FDG PET/CT radiomics-based ML analyses to address issues in classification or to construct prediction models for several types of tumors.

## Literature search and screening

On April 20, 2023, we searched studies with the following terms in the title from PubMed: “PET/CT” and “radiomic” or “radiomics” and “machine learning.”

**Fig. 1:**
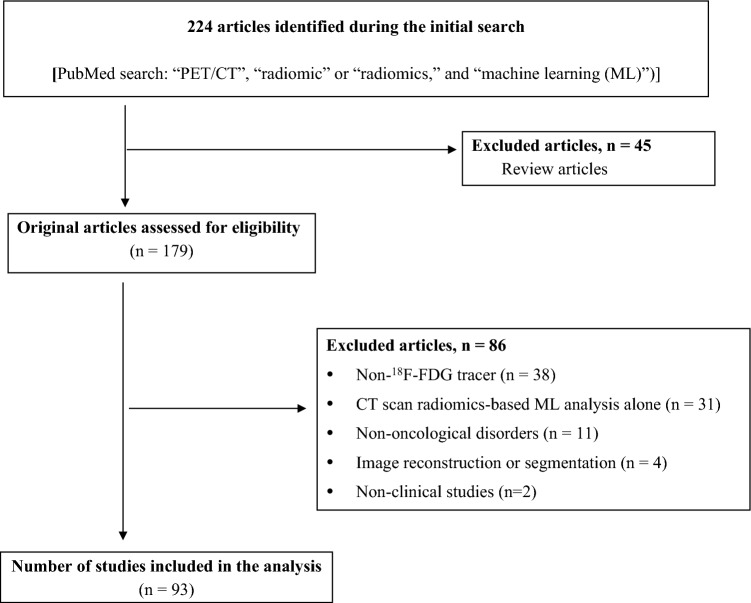
Flowchart of study retrieval via literature search and inclusion in the analysis

In total, 224 articles were identified during the initial search. The titles, abstracts, and texts were assessed to identify relevant articles. The inclusion criteria were as follows: (1) studies written in English, (2) original clinical studies about oncology, and (3) studies describing the application of the ^18^F-FDG PET/CT radiomics-based ML approach for solving issues associated with classifying or constructing prediction models. The exclusion criteria were as follows: 1) reports only describing the CT radiomics-based ML approach, 2) studies using ML for image reconstruction or segmentation, 3) cohort studies with < 20 patients, and 4) review articles. Of 224 articles identified, 45 were review articles; hence, they were not included in the study. Among the remaining 179 original articles, 86 were excluded because of non-^18^F-FDG tracer (*n* = 38), only CT-based radiomic ML analysis (*n* = 31), non-oncological disorders (*n* = 11), application of ML for image reconstruction or image segmentation (*n* = 4) and nonclinical studies (*n* = 2). Finally, 93 articles were included in the analysis, and all articles were published after 2018 (Fig. 1).

## Clinical application of ^18^F-FDG PET/CT radiomics-based ML analyses in lung or mediastinal tumors

### Difference between benign and malignant tumors and between primary and metastatic tumors

Pulmonary nodules are common clinical findings, and lung cancer frequently presents as a solitary pulmonary nodule (SPN) on diagnostic imaging at the early disease stage [13]. SPNs are often incidentally detected. Thus, benign SPNs should be clinically differentiated from malignant SPNs.

Ren et al. [14] reported that the ML model with the least absolute shrinkage and selection operator (LASSO) regression algorithm using combined clinical data and PET-radiomics had a good diagnostic performance for distinguishing benign from malignant SPNs, with an area under the receiver operating characteristic curve (AUC) of 0.94. Zhou et al. [15] examined the ability of ^18^F-FDG PET/CT radiomics-based ML analysis in differentiating primary from metastatic lung lesions. Results showed that the ML model with the gradient boosting decision tree algorithm with PET-radiomics had the highest classification accuracy, with an AUC of 0.983. Some studies have found similar results [16–19] (Table 1). Thus, ^18^F-FDG PET/CT radiomics-based ML analysis can have a great potential in characterizing SPNs.

**Table 1:** Summary of representative studies on ^18^F-FDG PET/CT radiomics-based machine learning analyses in lung and mediastinal tumors

Authors	Year	Tumor type	Aim	Sample size	Constructed ML models	Core ML algorithm	Best ML model	Validation	Result^a^
**Differentiating benign from malignant tumors or primary from metastatic tumors**									
Ren et al. [14]	2022	SPN	Benign vs. malignant	*n* = 280	Clinical model PET radiomics-based model Combined model	LASSO regression	Combined model	Training and validation cohorts	AUC: 0.94
Zhou et al. [15]	2021	SPN	Primary vs. metastatic	*n* = 769	CT radiomics-based model PET radiomics-based model	GBDT	PET radiomics-based model	Training and validation cohorts	AUC: 0.983
Salihoğlu et al. [16]	2022	SPN	Benign vs. malignant	*n* = 48	PET radiomics-based model alone	Deep neural network	–	Internal validation (cross-validation)	AUC: 0.81
Zhang et al. [17]	2019	SPN	Benign vs. malignant	*n* = 135	CT radiomics-based model PET radiomics-based model Combined model	SVM	Combined model	Internal validation (cross-validation)	AUC:0.887
Yan et al. [18]	2020	SPN	Primary vs. metastatic	*n* = 445	CT radiomics-based model PET radiomics-based model Combined model	SMO	Combined model	Internal validation (cross-validation)	AUC: 0.98
Agüloğlu et al. [19]	2023	Consolidated lesion	Lung cancer vs. infection	*n* = 106	PET radiomics-based model only	LR	–	Training and validation cohorts	AUC: 0.813
**Classifying tumors according to histological subtypes**									
Zhao et al. [22]	2022	NSCLC	ADC vs. SCC	*n* = 120	Clinical model PET radiomics-based model Combined model	SVM	Combined model	Training and validation cohorts	AUC: 0.876
Han et al. [23]	2021	NSCLC	ADC vs. SCC	*n* = 1419	PET radiomics-based model only	VGG16 DL	–	Training and validation cohorts	AUC: 0.903
Ren et al. [24]	2021	NSCLC	ADC vs. SCC	*n* = 315	Clinical laboratory model CT radiomics-based model PET radiomics-based model Combination of all models	LASSO regression	Combined model	Training and validation cohorts	AUC: 0.901
Koyasu et al. [25]	2020	NSCLC	ADC vs. SCC	*n* = 188	Combined CT + PET radiomics-based model alone	XGB	–	Internal validation (cross-validation)	AUC: 0.843
Hyun et al. [26]	2019	NSCLC	ADC vs. SCC	*n* = 396	Combined clinical + PET radiomics-based model alone	LR	–	Internal validation (cross-validation)	AUC: 0.859
Nakajo et al. [27]	2022	TET	Thymic carcinoma vs thymoma	*n* = 79	Combined PET radiomics- + CNN-based feature model	LR	–	Internal validation (cross-validation)	AUC: 0.90
Ozkan et al. [28]	2022	TET	Low-risk thymoma vs. high-risk thymoma	*n* = 27	Combined clinical + PET radiomics-based model alone	LASSO + artificial neural network	–	Training and validation cohorts	AUC: 0.88
**Predicting tumor characteristics**									
Gao et al. [33]	2023	Lung ADC	EGFR status	*n* = 515	Clinical model CT radiomics-based model PET radiomics-based model Combined models	RF	Combined model	Training and validation cohorts	AUC: 0.730
Chang et al. [34]	2021	Lung ADC	ALK status	*n* = 526	CT radiomics-based model PET radiomics-based model Combined PET and CT radiomics-based model Combined clinical, PET, and CT models	LASSO regression	Combined clinical, PET and CT model	Training and validation cohorts	AUC: 0.88
Shiri et al. [35]	2020	NSCLC	EGFR and KRAS status	*n* = 150	Combined CT + PET radiomics-based model alone	Stochastic gradient descent	–	Training and validation cohorts	AUC for EGFR: 0.82 AUC for KRAS: 0.83
Liu et al. [36]	2020	Lung ADC	EGFR status	*n* = 148	Combined CT + PET radiomics-based model alone	XGB	–	Training and validation cohorts	AUC: 0.870
Agüloğlu et al. [37]	2022	NSCLC	EGFR and ALK status	*n* = 189	PET radiomics-based model alone	Naïve Bayes algorithm	–	Training and validation cohorts	AUC for EGFR: 0.797 AUC for ALK: 0.814
Nair et al. [38]	2021	NSCLC	EGFR status	*n* = 50	CT radiomics-based model PET radiomics-based model	LR	PET-radiomics model	Internal validation (cross-validation)	AUC: 0.870
Li. et al. [39]	2019	NSCLC	EGFR status	*n* = 115	CT radiomics-based model PET radiomics-based model Combined model	LASSO regression	Combined model	Internal validation (cross-validation)	AUC: 0.822
Lim et al. [40]	2022	NSCLC	PD-L1 expression	*n* = 312	Combined model only (CT + PET radiomics feature)	Naïve Bayes algorithm	–	Internal validation (cross-validation)	AUC: 0.712
Mu et al. [41]	2021	NSCLC	PD-L1 expression	*n* = 697	Combined CT + PET radiomics-based model alone	SRecCNN	–	Training and validation cohorts	AUC: 0.82
Tong et al. [42]	2022	NSCLC	CD8 expression	*n* = 1367	CT radiomics-based model PET radiomics-based model Combined PET and CT scan model Combined clinical, PET, and CT scan model	LR	Combined clinical, PET and CT model	Training and validation cohorts	AUC: 0.932
**Predicting tumor stage**									
Wang et al. [44]	2023	NSCLC	N stage	*n* = 192	Combined clinical, tumor PET, and tumor CT model Combined clinical, lymph node PET, and lymph node CT model Combination of all models	XGB	Combination of all models	Training and validation cohorts	N2 stage, AUC: 0.94
Laros et al. [45]	2022	NSCLC	LNM	*n* = 148	Combined tumor and lymph node PET radiomics-based model alone	XGB	–	Training and validation cohorts	Accuracy: 0.88
Onozato et al. [46]	2023	Lung cancer	Highly invasive lung cancer	*n* = 873	CT radiomics-based model PET radiomics-based model Combined model	Ensemble ML algorithm	Combined model	Training and validation cohorts	AUC: 0.880
**Predicting treatment response or survival**									
Zhao et al. [47]	2022	Lung ADC	OS	*n* = 421	Combined clinical + CT radiomics-based + PET radiomics-based model alone	Ensemble ML algorithm	–	Training and validation cohorts	3-year OS, AUC: 0.84; 4-year OS, AUC: 0.88
Huang et al. [48]	2022	Malignant lung tumor	OS	*n* = 965	Clinical model CT radiomics-based model PET radiomics-based model Combined PET and CT scan model Combined clinical, PET, and CT scan model	CNN + RSF	Combined clinical, PET, and CT scan models	Training and validation cohorts	C-index: 0.737
Ahn et al. [49]	2019	NSCLC	Disease recurrence after surgery	*n* = 93	PET radiomics-based model alone	RF	–	Training and validation cohorts	AUC: 0.956
Kirienko et al.[50]	2021	NSCLC	Disease recurrence after surgery	n = 151	Genomic model Combined PET and CT model Combination of all models	Logic learning machine	Combination of all models	Internal validation (cross-validation)	AUC: 0.87
Mu et al.[51]	2020	NSCLC	PFS in patients treated with EGFR-TKI	*n* = 616	Combined CT + PET radiomics-based model alone	SRecCNN	–	Training and validation cohorts	HR: 0.24
Mu et al. [52]	2020	NSCLC	PFS and OS in patients treated with ICI	*n* = 194	Combined CT radiomics-based + PET radiomics-based + PET/CT scan-based (minimum Kullback–Leibler divergence features) model alone	LASSO + Cox proportional hazard model	–	Training and validation cohorts	PFS, C-index: 0.77; OS, C-index: 0.80
Bertolini.et al. [53]	2022	NSCLC	2-year PFS in patients treated with RT	*n* = 117	Harmonized CT radiomics-based model Harmonized PET radiomics-based model Combined model	SVM	Combined model	Training and validation cohorts	AUC: 0.77
Sepehri et al. [54]	2021	NSCLC	OS in patients treated with CRT	n = 138	Combined CT + PET radiomics-based model alone	Ensemble ML algorithm	–	Training and validation cohorts	Accuracy: 0.78
Afshar et al. [55]	2020	NSCLC	OS in patients treated with RT	*n* = 132	Combined clinical + CT radiomics-based + PET radiomics-based model alone	CNN + Cox proportional hazard model	–	Training and validation cohorts	C-index: 0.68
Astaraki et al. [56]	2019	NSCLC	OS in patients treated with CRT	*n* = 30	CT radiomics-based model PET radiomics-based model Combined model	SVM	Combined model	Internal validation (cross-validation)	AUC: 0.95
Park et al. [57]	2023	NSCLC	Disease recurrence in patients treated with surgery or RT	*n* = 77	Combined clinical + PET radiomics-based model alone	Naïve Bayes algorithm	–	Training and validation cohorts	AUC: 0.816
Pavic et al. [58]	2020	MPM	PFS in patients treated with surgery	*n* = 72	CT radiomics-based model PET radiomics-based model	PCA + cox proportional hazard model	PET radiomics-based model	Training and validation cohorts	C-index: 0.66

*ADC* adenocarcinoma, *ALK* anaplastic lymphoma kinase, *AUC* area under the receiver operating characteristic curve, *C-index* concordance index, *CNN* convolutional neural network, *CRT* chemoradiotherapy, *DL* deep learning, *EGFR* epidermal growth factor receptor, *GBDT* gradient boosting decision tree, *HR* hazard ratio, *ICI* immune checkpoint inhibitor, *KRAS* kirsten rat sarcoma viral oncogene, *LASSO* least absolute shrinkage and selection operator algorithm, *LNM* lymph node metastasis, *LR* logistic regression, *ML* machine learning, *MPM* malignant pleural mesothelioma, *NSCLC* non-small cell lung cancer, *OS* overall survival, *PCA* principal component analysis, *PD-L1* programmed death ligand, *PFS* progression-free survival, *RF* random forest, *RSF* random survival forests, *RT* radiotherapy, *SCC* squamous cell carcinoma, *SMO* sequential minimal optimization, *SPN* solitary pulmonary nodule, *SRecCNN* small-residual-convolutional-network, *SVM* support vector machine, *TET* thymic epithelial tumor, *TKI* tyrosine kinase inhibitor, *XGB* gradient tree boosting

^a^Performance only presents the result of the best machine learning model

### Classification according to histological types

Due to the different histologic and biological characteristics of lung adenocarcinoma (ADC) and lung squamous cell carcinoma (SCC), their treatment regimen, prognosis, and relapse rate significantly vary [20, 21]. Thus, it is important to distinguish these two subtypes of non-small cell lung cancer (NSCLC) before treatment for appropriate clinical decision-making.

^18^F-FDG PET/CT radiomics-based ML analysis might improve the classification of ADC and SCC [22–26]. Zhao et al. [22] established combined ML models based on clinical characteristics (sex and smoking status), laboratory findings (carcinoembryonic antigen and squamous cell carcinoma antigen levels), and PET-radiomics to classify ADC and SCC. The support vector machine (SVM) algorithm accurately distinguished ADC from SCC, with an AUC of 0.876. This algorithm had a significantly better prediction performance than the clinical model (AUC:0.712, *p* = 0.037). Han et al. [23] examined the usefulness of PET radiomics-based ML/DL algorithms for obtaining differential diagnosis in patients with ADC and SCC. They reported that ML analyses with either the linear discriminant analysis (AUC: 0.863) or the SVM (AUC: 0.863) algorithm had optimal performance. Moreover, the VGG16 DL algorithm (AUC: 0.903) outperformed all conventional ML algorithms. Similar studies have successfully differentiated ADC from SCC [24–26] (Table 1).

^18^F-FDG PET/CT radiomics-based ML analysis can characterize histological subtypes in thymic epithelial tumors (TETs) [27, 28]. The ML model trained using ^18^F-FDG PET radiomics and DL-based features with the logistic regression (LR) algorithm was proposed for predicting the histological subtypes of TETs [27]. This model can accurately differentiate thymic cancer from thymoma, with an AUC of 0.90.

### Prediction of tumor characteristics

Recently, the treatment options for NSCLC significantly improved with advancements in targeted therapies against mutated genes such as epidermal growth factor receptor (EGFR), kirsten rat sarcoma viral oncogene (KRAS), and anaplastic lymphoma kinase (ALK) [29, 30]. Moreover, immune checkpoint inhibitors targeting programmed cell death protein 1 (PD-1) or programmed death ligand 1 (PD-L1) are associated with better survival outcomes compared with conventional chemotherapy in patients with advanced-stage NSCLC [31, 32]. Thus, in patients with NSCLC, gene mutations or the immune checkpoint status of tumors should be identified to determine the appropriate treatment strategy.

Several reports have examined the usefulness of ^18^F-FDG PET/CT radiomics-based ML analysis for predicting gene mutation. Previous studies commonly showed that ^18^F-FDG PET/CT radiomics-based ML analysis had a promising performance for predicting gene mutation [33–39] (Table 1). Gao et al. [33] constructed radiomics-based models based on ^18^F-FDG PET/CT features using ML to predict EGFR mutation status in patients with lung ADC. Results showed that the ML model with the random forest (RF) algorithm using combined clinical data, CT-radiomics and PET-radiomics had the highest performance, with an AUC of 0.730. Chang et al. [34] revealed that the combined clinical data and PET/CT-based ML model with the LASSO regression algorithm is significantly advantageous in predicting ALK mutation status in patients with lung ADC compared with the clinical model (AUC:0.88 vs. 0.74, *p* < 0.001). Shiri et al. [35] reported that the ML model with the stochastic gradient descent algorithm using CT-radiomics and PET-radiomics outperformed conventional methods (peak of standardized uptake value [SUVpeak] or metabolic tumor volume [MTV]) in predicting EGFR and KRAS gene mutation status in NSCLC (EGFR: SUVpeak [AUC: 0.69] vs. ML model [AUC: 0.82]; KRAS: MTV [AUC: 0.55] vs. ML model [AUC: 0.83]). Previous studies have shown that the ^18^F-FDG PET/CT radiomics-based ML model have a similar performance, with AUCs of 0.797–0.870 [36–39].

Several studies have assessed the predictive ability of ^18^F-FDG PET/CT radiomics-based ML analysis for immune checkpoint status in NSCLC [40–42]. Lim et al. [40] predicted the PD-L1 expression level in patients with NCSLC using the ^18^F-FDG PET/CT radiomics-based ML model. Results showed that the ML model with the Naïve Bayes algorithm using the top five features (CT_gray-level run length matrix [GLRLM]_long run high grey-level emphasis, CT_grey-level co-occurrence matrix [GLCM]_homogeneity, CT_mean Hounsfield unit, CT_GLRLM_long run emphasis, and PET_SUVmax) had the best predictive performance (AUC: 0.712). Mu et al. [41] developed a ^18^F-FDG PET/CT-based DL model to evaluate PD-L1 status. Results showed that the deep learning score (DLS) could significantly distinguish PD-L1-positive from PD-L1-negative patients (AUC: 0.82).

### Predicting tumor stage

The clinical outcome of NSCLC is directly related to its stage at diagnosis [43]. Moreover, there were reports showing the usefulness of the ^18^F-FDG PET/CT radiomics-based ML method for predicting tumor stage in lung cancer [44–46]. Wang et al. [44] reported that the ML model with the gradient tree boosting (XGB) ML algorithm using combined clinical data and PET/CT radiomics of the primary tumor and lymph node had the highest diagnostic performance in predicting lymph node metastasis (LNM) in NSCLC (AUC: 0.93). Moreover, this model had a great potential in predicting N2 stage NSCLC (AUC: 0.94). In addition, Laros et al. [45] reported that the combined PET-radiomics of the primary tumor and lymph node had good performance in predicting LNM from NSCLC, with an accuracy of 0.88.

### Predicting treatment response or survival

Previous studies have examined the potential of ML analysis using pretreatment ^18^F-FDG PET/CT radiomic features for predicting patient response and survival in malignant lung tumors [47–57] (Table 1).

Zhao et al. [47] examined the ability of ML models trained using clinical data and ^18^F-FDG PET/CT radiomics for predicting overall survival (OS) in patients with lung ADC who underwent surgery and received radiotherapy (RT), chemotherapy, or immunotherapy. The ensemble ML models, which were constructed with clinical data and ^18^F-FDG PET/CT radiomic features, could predict the 3- and 4-year OS, with an AUC of 0.84 and 0.88, respectively. Huang et al. [48] showed that the convolutional neural networks (CNNs) trained by ^18^F-FDG PET/CT had good performance in predicting OS in patients with malignant lung tumor who received RT, chemotherapy, or immunotherapy. To predict OS, the CNNs trained using clinical data and ^18^F-FDG PET/CT radiomics with the random survival forest (RSF) ML model (concordance index [C-index]: 0.737) had a similar performance to CT alone (C-index: 0.730). However, it had a better performance than PET (C-index: 0.595) and clinical models (C-index: 0.595) alone.

Previous studies have assessed the ability of ^18^F-FDG PET/CT radiomics-based ML models for predicting outcomes in not only patients with surgically treated NSCLC [49, 50] but also those with nonsurgically treated NSCLC [51–56]. Ahn et al. [49] used the ^18^F-FDG PET/CT radiomics-based ML approach to predict disease recurrence in patients with NSCLC who underwent surgery. Results showed that the ML model with the RF algorithm had good performance for predicting recurrence, with an AUC of 0.956. Mu et al. [51] established the ^18^F-FDG PET-based DLSs, which is useful for predicting EGFR mutation status (EGFR-DLS) (AUC: 0.81). EGFR-DLS was significantly and positively associated with a longer progression-free survival (PFS) in patients treated with EGFR-tyrosine kinase inhibitors (hazard ratio [HR]:0.24, *p* < 0.001). Mu et al. [52] reported that the ^18^F-FDG PET/CT radiomics-based ML model had a good AUC for predicting response to immune checkpoint inhibitors (0.81). Moreover, the constructed nomogram models (C-indices of 0.77 and 0.80 for predicting OS and PFS, respectively) had good performance in predicting prognosis. Similar studies have successfully predicted treatment responses or survival in patients with NSCLC [50, 53–57] (Table 1).

The ^18^F-FDG PET/CT radiomics-based ML analysis has been applied to predict PFS in malignant pleural mesothelioma [58]. This study showed the prognostic potential of the cox regression ML model established using specific PET radiomics-based on the principal component analysis for PFS with a C-index of 0.66.

## Summary

Previous studies commonly showed that ^18^F-FDG PET radiomics-based ML analysis had a high predictive performance for differentiating benign from malignant tumors, predicting tumor characteristics, staging tumors, and assessing treatment outcome or prognosis in lung or mediastinal tumors, with AUCs, accuracies, or C-indices of > 0.70. Thus, the ^18^F-FDG PET radiomics-based ML analysis might play important roles in supporting clinicians in diagnostic and patient management including precision medicine for lung or mediastinal tumors. However, as shown in Table 1, previous studies have reported several ML processes including ML algorithms, and different ML models have been applied for the same purpose.

## Clinical application of ^18^F-FDG PET/CT radiomics-based ML analyses in head and neck tumors

### Differentiating benign and malignant tumors and predicting tumor characteristics

In head and neck tumors, ^18^F-FDG PET/CT radiomics-based ML analyses have been applied to differentiate benign from malignant tumors or to predict tumor characteristics. The following articles have reported about differentiating benign from malignant tumors.

In thyroid incidentalomas, distinguishing benign from malignant tumors based on SUVmax on ^18^F-FDG PET/CT is challenging due to a significant overlap between these lesions [59]. Aksu et al. [60] reported that the ML model with the RF algorithm had a better performance in differentiating benign from malignant thyroid incidentalomas based on SUVmax (AUC: 0.849 vs. 0.758).

The assessment of human papillomavirus (HPV) status plays an important role in treatment planning for oropharyngeal cancer [61]. Haider et al. [62] showed that the AUC of combined tumor and lymph node PET/CT radiomics-based ML model with the XGB algorithm for predicting HPV status in oropharyngeal cancer was 0.83.

### Predicting treatment response or survival

Previous studies have reported the predictive ability of ^18^F-FDG PET/CT radiomics-based ML analysis for treatment outcomes in head and neck cancers [63–71] (Table 2). Haider et al. [63] showed that the ML model with the RSF algorithm using clinical and pretreatment ^18^F-FDG PET/CT radiomics had good predictive performance for locoregional progression in patients with HPV-associated oropharyngeal cancer who received RT (C-index: 0.76). In hypopharyngeal cancers, the ML model with the LR algorithm constructed based on UICC stage, T and N stage, and pretreatment ^18^F-FDG PET-radiomics with GLCM_entropy and GLRLM_ run length non-uniformity (RLNU) is a significant predictor of PFS (HR:3.22, *p* = 0.045) [64].

**Table 2:** Summary of representative studies on ^18^F-FDG PET/CT radiomics-based machine learning analyses in head and neck tumors

Authors	Years	Tumor type	Aim	Sample size	Constructed ML models	Core ML algorithm	Best ML model	Validation	Results^a^
**Differentiating benign from malignant tumors**									
Aksu et al. [60]	2020	Thyroid incidentaloma	Benign vs. malignant	*n* = 60	PET radiomics only	RF	–	Training and validation cohorts	AUC: 0.849
**Predicting tumor characteristics**									
Haider et al. [62]	2020	OPC	HPV status	*n* = 435	Tumor PET/CT Lymph node PET/CT Tumor and lymph node PET/CT	XGB	Tumor and lymph node PET/CT	Training and validation cohorts	AUC: 0.83
**Predicting treatment response or survival**									
Haider et al. [63]	2021	OPC	Locoregional recurrence after RT	*n* = 190	Clinical model CT radiomics-based model PET radiomics-based model Combined PET and CT model Combined clinical, PET, and CT model	RSF	Combined model	Internal validation (cross-validation)	C-index: 0.76
Nakajo et al. [64]	2023	HPC	PFS after RT, CRT, or surgery	*n* = 100	Combined clinical + PET radiomics-based model alone	LR	–	Training and validation cohorts	HR: 3.22
Lafata. et al. [65]	2021	OPC	Recurrence-free survival after RT	*n* = 64	Intra-treatment PET radiomics-based model	Unsupervised data clustering algorithm	–	Internal validation	HR: 2.69
Spielvogel et al. [66]	2023	HNSCC	3-year OS	*n* = 127	Combined genomic + CT radiomics-based + PET radiomics-based model alone	Ensemble ML algorithm	–	Internal validation (cross-validation)	AUC: 0.75
Haider et al. [67]	2020	OPC	OS after RT, CRT, or surgery	*n* = 306	Clinical model CT radiomics-based model PET radiomics-based model Combined PET and CT model Combined clinical, PET, and CT model	RSF	Combined model	Training and validation cohorts	5-year OS, HPV-associated oropharyngeal cancer (*p* = 0.02); 5-year OS, HPV-negative oropharyngeal cancer (*p* = 0.01)
Zhong et al. [68]	2021	HPC and LC	Disease progression at 1 year after chemotherapy or RT	*n* = 72	CT radiomics-based model PET radiomics-based model Combined model	RF	Combined model	Training and validation cohorts	AUC: 0.94
Du et al. [69]	2019	NPC	Local recurrence after chemotherapy or RT	*n* = 76	PET radiomics-based model alone	RF	–	Internal validation (cross-validation)	AUC: 0.892
Peng et al. [70]	2019	NPC	5-year DFS after chemotherapy or CRT	*n* = 707	Combined PET radiomics-based + CNN-based model alone	LASSO regression	–	Training and validation cohorts	C-index: 0.722
Liu et al. [71]	2020	HNSCC	OS after RT	*n* = 171	PET radiomics-based model alone	LASSO regression	–	Internal validation (cross-validation)	C-index: 0.77

^a^Performance only presents the result of the best machine learning model

*AUC* area under the receiver operating characteristic curve, *C-index* concordance index, *CNN* convolutional neural network, *CRT* chemoradiotherapy, *DFS* disease-free survival, *HNSCC* head and neck squamous cell carcinoma, *HPC* hypopharyngeal cancer, *HPV* human papillomavirus, *HR* hazard ratio, *LASSO* least absolute shrinkage and selection operator algorithm, *LC* laryngeal cancer, *LR* logistic regression, *ML* machine learning, *NPC* nasopharyngeal cancer, *OPC* oropharyngeal cancer, *OS* overall survival, *PFS* progression-free survival, *RF* random forest, *RSF* random survival forest, *RT* radiotherapy, *XGB* gradient tree boosting

Previous studies have reported the usefulness of intra-treatment ^18^F-FDG PET/CT radiomics-based ML analysis for outcome prediction in head and neck cancers. Lafata et al. [65] showed that the unsupervised clustering of intra-treatment ^18^F-FDG PET/CT radiomics, which were obtained 2 weeks after RT (at a dose of 20 Gy), was significantly associated with recurrence-free survival (HR:2.69, *p* = 0.04) in patients with oropharyngeal cancer who received definitive RT. Moreover, a previous study assessed the ability of ML analysis using the combined ^18^F-FDG PET radiomics and genomic data for predicting 3-year OS in head and neck cancers (AUC: 0.75) [66]. Similar studies have successfully predicted prognosis in head and neck cancer [67–71] (Table 2).

## Summary

Previous studies revealed that ^18^F-FDG PET/CT radiomics-based ML analysis had good predictive performances for predicting treatment outcome or prognosis, with AUCs or C-indices of > 0.70, in head and neck tumors. Thus, ^18^F-FDG PET/CT radiomics-based ML analysis might be expected to be an important tool for patient management in head and neck tumors. However, several ML processing approaches have also been discussed (Table 2).

## Clinical application of ^18^F-FDG PET/CT radiomics-based ML analyses in lymphatic tumors

### Differentiating benign from malignant tumors and primary from metastatic tumors or classifying tumors according to histological types

The conventional semi-quantitative ^18^F-FDG PET parameters such as SUVmax, MTV, and total lesion glycolysis (TLG) are useful biomarkers for characterizing malignant lymphoma [72, 73]. However, the ability of these parameters in identifying tumor heterogeneity, which ultimately contributes to tumor aggressiveness and poor prognosis, remains limited [74]. Recently, ^18^F-FDG PET/CT radiomics-based ML analysis has been applied to overcome these issues [75]. Previous studies have revealed that ^18^F-FDG PET/CT radiomics-based ML analysis is useful in not only classifying tumors based on histological subtypes but also differentiating malignant lymphoma from other diseases [76–80].

Abenavoli et al. [76] showed that the ML model with the RF algorithm using PET-radiomics had a better performance in differentiating diffuse large B-cell lymphoma (DLBCL) from Hodgkin’s lymphoma (HD) based on SUVmax (AUC: 0.87 vs. 0.78). de Jesus et al. [77] reported that the ML model with the gradient boosting algorithm using PET/CT radiomics had a significantly higher AUC in distinguishing DLBCL and follicular lymphoma according to SUVmax (AUC:0.86 vs. 0.79, *p* < 0.01). Lovinfosse et al. [78] also showed that the ML model with the RF algorithm using clinical data and PET-radiomics had good performance in differentiating DLBCL from HD, with an AUC of 0.95. Further, the authors showed that the constructed ML model with the RF algorithm had good performance in differentiating malignant lymphoma and sarcoidosis, with an AUC of 0.94. Yang et al. [79] revealed that the ML model with the SVM algorithm constructed according to combined CNN-based features and PET-radiomics had a great potential in distinguishing malignant lymphoma from enlarged metastatic cervical lymph nodes (AUC: 0.948).

### Predicting treatment response or survival

For the treatment assessment of malignant lymphoma, the visual assessment of the Deauville score (DC) has been a useful ^18^F-FDG PET/CT criterion: DC1–DC3, complete metabolic response; DC4 and DC5, incomplete metabolic response [81–83]. However, there might be difficulties in predicting treatment outcomes based on DC alone because of the inter- or intra-variability of DC definition. Thus, ^18^F-FDG PET/CT radiomics-based ML analysis can be a novel approach for predicting treatment outcomes in malignant lymphoma.

Frood et al. [84] examined the ability of pretreatment ^18^F-FDG PET/CT radiomics-based ML analysis for predicting recurrence after DLBCL treatment. Results showed that the ML model with the ridge regression algorithm using combined clinical and PET-radiomics had good performance, with an AUC of 0.73. Cui et al. [85] assessed the potential of the ^18^F-FDG PET/CT radiomics-based ML approach for identifying patients with DLBCL who are at high risk for progression or relapse after receiving first-line therapy. Results showed that the ML model with the RF algorithm using clinical data, baseline, end-of-treatment and delta PET-radiomics features was a significant predictor of PFS (C-index: 0.853). By contrast, ^18^F-FDG PET/CT radiomics-based ML analysis was found to be useful for predicting recurrence after HD treatment [86, 87]. Frood et al. [86] showed that the ML model with the ridge regression algorithm using combined clinical data and PET-radiomics had good predictive performance, with an AUC of 0.81. Similar studies have successfully predicted treatment responses or survival in malignant lymphoma [87–91] (Table 3).

**Table 3:** Summary of representative studies on ^18^F-FDG PET/CT radiomics-based machine learning analyses in lymphatic tumors

Authors	Years	Tumor type	Aim	Sample size	Constructed ML models	Core ML algorithm	Best ML model	Validation	Results^a^
**Differentiating benign from malignant tumors and primary from metastatic tumors or classifying tumors according to pathological subtypes**									
Abenavoli et al. [76]	2023	Malignant lymphoma	DLBCL vs. HD	*n* = 117	PET radiomics-based model alone	RF	–	Training and validation cohorts	AUC: 0.87
de Jesus et al. [77]	2022	Malignant lymphoma	DLBCL vs. FL	*n* = 120	Combined CT + PET radiomics-based model alone	Gradient boosting	–	Training and validation cohorts	AUC: 0.86
Lovinfosse et al. [78]	2022	Malignant lymphoma	1. Malignant lymphoma vs. sarcoidosis 2. DLBCL vs. HD	*n* = 420	Combined clinical + PET radiomics-based model alone	RF	–	Training and validation cohorts	1. AUC: 0.94 2. AUC: 0.95
Yang et al. [79]	2023	Cervical lymph node	Malignant lymphoma vs. metastasis	*n* = 165	CNN model Combined PET radiomics-based + CNN-based model alone	SVM	Combined model	Training and validation cohorts	AUC: 0.948
Cui et al. [80]	2023	Brain tumor	Malignant lymphoma vs. metastasis	*n* = 51	PET radiomics-based model alone	RF	–	Training and validation cohorts	AUC: 0.93
**Predicting treatment response or survival**									
Frood et al. [84]	2022	DLBCL	Recurrence after chemotherapy	*n* = 229	Combined clinical + PET radiomics-based model alone	Ridge regression	–	Training and validation cohorts	AUC: 0.73
Cui et al. [85]	2022	DLBCL	PFS after chemotherapy	*n* = 271	Clinical model PET radiomics-based model Combined clinical + PET radiomics-based model alone	RF + cox proportional hazard	Combined model	Training and validation cohorts	C-index: 0.853
Frood et al. [86]	2022	HD	Recurrence after chemotherapy or RT	*n* = 289	Combined clinical + PET radiomics-based model alone	Ridge regression	–	Training and validation cohorts	AUC: 0.81
Ritter et al. [87]	2022	DLBCL	Recurrence after chemotherapy	*n* = 85	PET radiomics-based model alone	Ensemble ML algorithm	–	Training and validation cohorts	AUC: 0.85
Jiang et al. [88]	2022	DLBCL	OS and PFS after chemotherapy	*n* = 383	Clinical model PET radiomics-based model Combined clinical + PET radiomics-based model alone	Ensemble ML algorithm	Combined model	Training and validation cohorts	PFS, C-index: 0.758, OS, C-index: 0.794,
Jiang et al. [89]	2022	GI DLBCL	OS and PFS after chemotherapy	*n* = 140	Clinical model Combined clinical + PET radiomics-based model alone	SVM + cox proportional hazard	Combined model	Training and validation cohorts	PFS, C-index: 0.831 OS, C-index: 0.877
Coskun et al. [90]	2021	DLBCL	Incomplete response after chemotherapy	*n* = 45	PET radiomics-based model alone	LR	–	Internal validation	AUC: 0.81
Milgrom et al. [91]	2019	HD	Recurrence after chemotherapy	*n* = 251	PET radiomics-based model alone	SVM with AdaBoost	–	Internal validation	AUC: 0.952

*AUC* area under the receiver operating characteristic curve, *C-index* concordance index, *CNN* convolutional neural network, *DLBCL* diffuse large B-cell lymphoma disease-free survival, *FL* follicular lymphoma, *GI* gastrointestinal, *HD* Hodgkin’s lymphoma, *LR* logistic regression, *ML* machine learning, *OS* overall survival, *PFS* progression-free survival, *RF* random forest, *RT* radiotherapy, *SVM* support vector machine

^a^Performance only presents the result of the best machine learning model

## Summary

Previous studies have shown that ^18^F-FDG PET/CT radiomics-based ML analysis is useful in not only differentiating but also predicting treatment outcome or prognosis in patients with malignant lymphomas. Each best ML model had good predictive performance, with AUCs or C-indices of > 0.70 (Table 3). Thus, it might be expected to promote the translation of ^18^F-FDG PET/CT radiomics-based ML analysis into clinical practice in the field of lymphatic tumors. However, the articles included in this review showed heterogeneity among various ML approaches.

## Clinical application of ^18^F-FDG PET/CT radiomics-based ML analyses in breast tumors

### Differentiating benign from malignant tumors and predicting tumor characteristics or stage

Several studies have examined the clinical potential of ^18^F-FDG PET/CT radiomics-based ML analyses in differentiating benign from malignant tumors and predicting tumor characteristics or stage in breast cancer [92–96].

Eifer et al. [92] showed that ML analyses with the k-nearest neighbors (kNN) algorithm using CT-radiomics and PET-radiomics had good performance in differentiating LNM from breast cancer from post-COVID-19 vaccine-associated axillary lymphadenopathy, with an AUC of 0.98.

An accurate assessment of both hormone receptor status and human EGFR 2 (HER2) status is important for treatment planning in breast cancer [97, 98]. Moreover, an accurate pretreatment assessment of axillary lymph node is essential in managing breast cancer [99]. Chen et al. [93] showed that the constructed ML model with the XGB algorithm based on PET/CTmean radiomics had good predictive ability for HER2 status in breast cancer (AUC: 0.76). In addition, Song [94] reported that the constructed ML model with the XGB algorithm based on PET/CT radiomics had good performance for predicting axillary LNM in patients with breast cancer (AUC: 0.890). A similar study has successfully predicted hormone status in breast cancer [95] (Table 4).

**Table 4:** Summary of representative studies on ^18^F-FDG PET/CT radiomics-based machine learning analyses in breast tumors

Authors	Years	Tumor type	Aim	Sample size	Constructed ML models	Core ML algorithm	Best ML model	Validation	Results^a^
**Differentiating benign from malignant tumors and predicting tumor characteristics or stage**									
Eifer et al. [92]	2022	Axillary LN	COVID-19 vaccine-associated lymphadenopathy vs. metastasis	*n* = 99	CT radiomics-based model PET radiomics-based model Combined model	kNN	Combined model	Training and validation cohorts	AUC: 0.98
Chen et al. [93]	2022	Breast cancer	HER2 status	*n* = 217	CT radiomics-based model PET radiomics-based model PET/CTconcat radiomics-based model PET/CTmean radiomics-based model	XGB	PET/CTmean radiomics model	Training and validation cohorts	AUC: 0.760
Song [94]	2021	Breast cancer	LNM	*n* = 100	PET radiomics-based model alone	XGB	–	Training and validation cohorts	AUC: 0890
Krajnc et al. [95]	2021	Breast cancer	Triple negative hormone status	*n* = 170	Combined clinical + CT radiomics-based + PET radiomics-based model alone	Ensemble ML algorithm	–	Internal validation (cross-validation)	AUC: 0.82
Ou et al. [96]	2020	Breast tumor	Breast cancer vs. malignant lymphoma	*n* = 44	SUV model CT radiomics-based model PET radiomics-based model Combined clinical + PET radiomics-based model Combined clinical + CT radiomics-based model	LASSO + LDA	Combined clinical and PET radiomics model	Training and validation cohorts	AUC: 0.806
**Predicting treatment response or survival**									
Li et al. [100]	2020	Breast cancer	pCR after NAC	*n* = 100	CT radiomics-based model PET radiomics-based model Combined age + CT radiomics-based + PET radiomics-based model	RF	Combined model	Training and validation cohorts	Accuracy: 0.80
Gómez et al. [101]	2022	Metastatic breast cancer	Metabolic response after treatment	*n* = 48	Combined clinical + CT radiomics-based + PET radiomics-based model alone	LASSO + SVM	–	Training and validation cohorts	AUC: 0.82

*AUC* area under the receiver operating characteristic curve, *HER2* human epidermal growth factor receptor, *kNN* k-nearest neighbors, *LASSO* least absolute shrinkage and selection operator algorithm, *LDA* linear discriminant analysis, *LN* lymph node, *LNM* lymph node metastasis, *ML* machine learning, *NAC* neoadjuvant chemotherapy, *pCR* pathological complete response, *RF* random forest, *SVM* support vector machine, *XGB* gradient tree boosting

^a^Performance only presents the result of the best machine learning model

### Predicting treatment response or survival

Two studies have examined the ability of ^18^F-FDG PET/CT radiomics-based ML analysis for predicting treatment outcome in breast cancer [100, 101]. Li et al. [100] assessed the usefulness of ^18^F-FDG PET/CT radiomics-based ML analysis for predicting pathological complete response (pCR) to neoadjuvant chemotherapy (NAC) in breast cancer. Results showed that the diagnostic accuracy of the ML model with the RF algorithm constructed based on patient age and PET/CT radiomics increased compared with that of the ML model with the RF algorithm constructed according to PET/CT radiomics only (0.800 vs. 0.767). The authors hypothesized that the finding can be attributed to the fact that younger patients had a higher pCR rate than older ones. Gómez et al. [101] assessed the predictive ability of ^18^F-FDG PET/CT radiomics-based ML analysis for metabolic response after metastatic breast cancer treatment. Results showed that the ML model with the LASSO + SVM algorithm using combined clinical data and PET-radiomics had good performance, with an AUC of 0.82.

## Summary

In breast tumors, each best ML model had good predictive performance for differentiating benign from malignant tumors and for predicting tumor characteristics and stage and treatment outcome, with AUCs or accuracies of > 0.70 (Table 4). The heterogeneity of ML approaches was also noted in the reported studies.

Although there have been several studies that have explored the usefulness of ^18^F-FDG PET/CT radiomics-based ML analysis associated with breast tumors, it might be expected in the ^18^F-FDG PET/CT radiomics-based ML analysis to be a novel tool to patient management for breast tumors.

## Clinical application of ^18^F-FDG PET/CT radiomics-based ML analyses in abdominal tumors

### Differentiating benign from malignant tumors and predicting tumor characteristics or stage

In abdominal tumors, the usefulness of ^18^F-FDG PET/CT radiomics-based ML analyses in differentiating benign and malignant tumors and predicting tumor characteristics or stage has been evaluated [102–108] (Table 5).

**Table 5:** Summary of representative studies on ^18^F-FDG PET/CT radiomics-based machine learning analyses in abdominal tumors

Authors	Years	Tumor type	Aim	Sample size	Constructed ML models	Core ML algorithm	Best ML model	Validation	Results^a^
**Differentiating benign from malignant tumors**									
Zhang et al. [102]	2019	Pancreatic tumor	AIP vs. PDAC	*n* = 251	CT radiomics-based model PET radiomics-based model Combined model	SVM	Combined model	Internal validation (cross-validation)	Accuracy: 0.850
Wei et al. [103]	2023	Pancreatic tumor	AIP vs. PDAC	*n* = 112	CT radiomics-based + PET radiomics-based model DL feature-based model Multidomain fusion model (radiomics + DL features)	VGG11 DL algorithm	Multidomain fusion model	Internal validation (cross-validation)	Accuracy: 0.901
**Predicting tumor characteristics or stage**									
Xing et al. [104]	2021	PDAC	Pathological grade	*n* = 149	CT radiomics-based model PET radiomics-based model Combined model	XGB	Combined model	Training and validation cohorts	AUC: 0.921
Jiang et al. [105]	2022	HCC or ICC	MVI	HCC: *n* = 76; ICC: *n* = 51	Clinical model CT radiomics-based model PET radiomics-based model Combined optimal PET and CT radiomics-based model Combined best clinical, PET radiomics-based, or CT radiomics-based model	RF	Combined best clinical and PET feature-based model	Training and validation cohorts	AUC for HCC: 0.88 AUC for ICC: 0.90
Liu et al. [106]	2021	Gastric cancer	LNM	*n* = 185	CT radiomics-based model PET radiomics-based model Combined model	Adaboost	Combined model	Training and validation cohorts	Accuracy: 0.852
He et al. [107]	2021	Colorectal cancer	LNM	*n* = 199	Combined CT + PET radiomics-based model	XGB	–	Training and validation cohorts	Accuracy: 0.7636
Li et al. [108]	2021	Colorectal cancer	MSI	*n* = 173	Combined clinical + CT radiomics-based + PET radiomics-based model alone	Adaboost	–	Training and validation cohorts	AUC: 0.828
**Predicting treatment response or survival**									
Toyama et al. [109]	2020	Pancreatic cancer	1-year survival after RT, CRT, or surgery	*n* = 161	PET radiomics-based model alone	RF	–	Internal validation (cross-validation)	HR for GLZLM_GLNU: 2.0
Liu et al. [110]	2023	Gastric cancer	HER2 status Progression after surgery	*n* = 90	Combined clinical + CT radiomics-based + PET radiomics-based model	Adaboost	–	Training and validation cohorts	Accuracy for HER2: 0.833 Accuracy for progression: 0.778
Lv et al. [111]	2022	Colorectal cancer	Recurrence-free survival after surgery	*n* = 196	Clinical model CT radiomics-based model PET radiomics-based model Combined model	RSF	Combined model	Training and validation cohorts	C-index for all patients: 0.780 C-index for patients with stage III disease: 0.820
Shen et al. [112]	2020	Rectal cancer	pCR after NCRT	*n* = 169	PET radiomics-based model alone	RF	–	Internal validation	Accuracy: 0.953
Agüloğlu et al. [113]	2023	Metastatic rectal cancer	2-year OS	*n* = 62	PET radiomics-based model alone	RF	–	Internal validation (cross-validation)	AUC: 0.843

*AIP* autoimmune pancreatitis, *AUC* area under the receiver operating characteristic curve, *C-index* concordance index, *CRT* chemoradiotherapy, *DL* deep learning, *GLNU* gray-level non-uniformity, *GLZLM* gray-level zone length matrix, *HCC* hepatocellular carcinoma, *HER2* human epidermal growth factor receptor, *HR* hazard ratio, *ICC* intrahepatic cholangiocarcinoma, *LNM* lymph node metastasis, *ML* machine learning, *MSI* microsatellite instability, *MVI* microvascular invasion, *NCRT* neoadjuvant chemoradiotherapy, *OS* overall survival, *pCR* pathological complete response, *PDAC* pancreatic ductal adenocarcinoma, *RF* random forest, *RSF* random survival forest, *RT* radiotherapy, *SVM* support vector machine, *XGB* gradient tree boosting

^a^Performance only presents the result of the best machine learning model

In pancreatic tumors, the ML model with the SVM algorithm using CT-radiomics and PET-radiomics has been a useful tool for differentiating autoimmune pancreatitis (AIP) and pancreatic ductal adenocarcinoma (PDAC), with an accuracy of 0.850 [102]. Moreover, this group established the multidomain fusion DL model using CT-radiomics, PET-radiomics, and DL features for differentiating AIP from PDAC [103]. Results showed that the accuracy of this DL model improved (0.901) compared with that of the formerly published ML model [102]. Xing et al. [104] assessed the ability of ^18^F-FDG PET/CT radiomics-based ML analysis for predicting the pathological grade of PDAC. Results showed that the ML model with the XGB algorithm using the combined CT-radiomics and PET-radiomics (AUC: 0.921) was better in predicting the pathological grade of PDAC than the CT-radiomics alone (AUC: 0.817) or the PET radiomics-based model alone (AUC: 0.771).

In liver tumors, Jiang et al. [105] assessed the usefulness of ^18^F-FDG PET radiomics-based ML analysis for predicting microvascular invasion (MVI) in hepatocellular carcinoma (HCC) and intrahepatic cholangiocarcinoma (ICC). Results showed that the constructed ML model with the RF algorithm using PET-radiomics and clinical features (cancer antigen 19–9 level or tumor stage) was useful for predicting MVI in either HCC (AUC: 0.88) or ICC (AUC: 0.90) [105].

Liu et al. [106] constructed a useful ML model with the Adaboost algorithm using CT-radiomics and PET-radiomics for predicting LNM in gastric cancer with an accuracy of 0.852. This model detected some metastatic lymph nodes that were missed on contrast-enhanced CT scan (19.6%). Thus, the constructed ML model might offer a potentially useful adjunct to the current staging approaches for gastric cancer. He et al. [107] showed that the ML model with the XGB algorithm using CT-radiomics and PET-radiomics was successful in classifying regional LNM from colorectal cancer, with an accuracy of 0.7636. This ML model was better in predicting LNM than lymph node status, as described in clinical ^18^F-FDG PET/CT scan reports (accuracy: 0.7091). Li et al. [108] reported that ^18^F-FDG PET/CT radiomics-based ML analysis was useful for predicting the microsatellite instability (MSI) status, which is an essential prognostic factor of colorectal cancer. Results showed that the constructed ML model with the Adaboost algorithm using two selected radiomic features (PET-Skewness and CT-RoomMeanSquared) had good predictive performance for MSI, with an AUC of 0.828.

### Predicting treatment response or survival

Several reports examined the usefulness of ^18^F-FDG PET/CT radiomics-based ML analyses for predicting treatment outcome in abdominal tumors [109–113]. These studies showed that the ^18^F-FDG PET/CT radiomics-based ML analyses were the power tools for predicting treatment response or prognosis.

Toyama et al. [109] revealed that PET-radiomics with gray-level zone length matrix (GLZLM)_gray-level non-uniformity (GLNU) was the most important feature on the ML model with the RF algorithm for predicting 1-year survival in pancreatic cancer, and multivariate analysis with Cox hazard regression revealed GLZLM_GLNU as the only statistically significant PET-radiomics for predicting 1-year survival (HR:2.0, *p* = 0.0094). Liu et al. [110] constructed the ML model with the Adaboost algorithm using clinical data, CT-radiomics and PET-radiomics for predicting HER2 expression status or disease progression in gastric cancer. The predictive accuracies of constructed ML model for HER2 expression status and disease progression were 83.3% and 77.8%, respectively. Lv et al. [111] developed the ML mode with the RSF algorithm using clinical data, CT-radiomics and PET-radiomics to predict recurrence-free survival in patients with colorectal cancer who received surgery, and revealed that the constructed ML model had good performance in predicting the prognosis (C-index for all patients, 0.780; C-index for stage III patients, 0.820). Shen et al. [112] constructed the ML model with the RF algorithm using PET-radiomics for predicting pCR after neoadjuvant chemoradiotherapy (CRT) in rectal cancer, and this ML model showed high predictive performance with an accuracy of 0.953. Moreover, the ability of ML model with the RF algorithm using PET-radiomics for predicting 2-year OS has also been reported in metastatic rectal cancer (2-year OS; AUC:0.843) [113].

## Summary

In abdominal tumors, each best ^18^F-FDG PET radiomics-based ML model had good predictive performance for differentiating benign and malignant tumors, predicting tumor characteristics, staging tumors, or assessing treatment outcome with AUCs, accuracies, or C-indices of > 0.70 (Table 5). The application of ^18^F-FDG PET radiomics-based ML analyses might be especially expected in the field of gastrointestinal cancers.

## Clinical application of ^18^F-FDG PET/CT radiomics ML analyses in gynecological tumors

### Predicting tumor stage

The expression of some protein molecules such as cyclooxygenase-2 (COX-2) is associated with LNM and lymphovascular space invasion (LVSI) in cervical cancer [114, 115]. Tumor budding (TB) is defined as a single neoplastic cell or cell cluster of up to four neoplastic cells at the invasive front of the tumor or within the tumor mass (intratumoral budding) [116]. Moreover, TB is associated with LNM, LVSI, and prognosis in cervical cancer [117]. Some investigators applied the ^18^F-FDG PET/CT radiomics-based ML models for predicting not only LNM or LVSI but also the expression of COX-2 or TB status in cervical cancer [118–121] (Table 6).

**Table 6:** Summary of representative studies on ^18^F-FDG PET/CT radiomics-based machine learning analyses in gynecological tumors

Authors	Years	Tumor type	Aim	Sample size	Constructed ML models	Core ML algorithm	Best ML model	Validation	Results^a^
**Predicting tumor stage**									
Lucia et al. [118]	2023	Cervical cancer	LNM	*n* = 178	Clinical model PET radiomics-based model Combined clinical and PET radiomics-based model Combat PET radiomics-based model Combined clinical and combat PET radiomics-based model	Neural network	Combat PET-radiomics model	Training and validation cohorts	AUC: 0.96
Zhang et al. [119]	2022	Cervical cancer	COX-2 status *N* status	*n* = 148	PET radiomics-based model alone	LASSO + LR	–	Training and validation cohorts	AUC for COX-2: 0.814 AUC for LNM: 0.817
Li et al. [120]	2021	Cervical cancer	LVSI	*n* = 112	PET radiomics-based model alone	LASSO + LR	–	Training and validation cohorts	AUC: 0.806
Chong et al. [121]	2021	Cervical cancer	ITB	*n* = 76	PET radiomics-based model alone	LASSO + SVM	–	Training and validation cohorts	AUC: 0.784
**Predicting treatment response or survival**									
Ferreira et al. [122]	2021	Cervical cancer	Disease progression after CRT	*n* = 158	Combined clinical + PET radiomics-based model	RF	–	Training and validation cohorts	AUC: 0.78
Nakajo et al. [123]	2022	Cervical cancer	PFS after RT, CRT, or surgery	*n* = 50	Combined clinical + PET radiomics-based model	Naïve base algorithm	–	Internal validation (cross-validation)	HR: 6.89
Nakajo et al. [124]	2021	Endometrial cancer	PFS and OS after RT, CRT, or surgery	*n* = 53	Combined clinical + PET radiomics-based model	kNN	–	Internal validation (cross-validation)	PFS—HR for coarseness: 0.65; OS—HR for coarseness: 0.52

*AUC* area under the receiver operating characteristic curve, *COX-2* cyclooxygenase-2, *CRT* chemoradiotherapy, *HR* hazard ratio, *ITB* intratumoral budding, *kNN* k-nearest neighbors, *LASSO* least absolute shrinkage and selection operator algorithm, *LNM* lymph node metastasis, *LR* logistic regression, *LVSI* lymphovascular space invasion, *ML* machine learning, *OS* overall survival, *PFS* progression-free survival, *RF* random forest, *RT* radiotherapy, *SVM* support vector machine

^a^Performance only presents the result of the best machine learning model

Lucia et al. [118] developed the ML model with the neural network algorithm using combat harmonized PET-radiomics acquired from the different PET scanners (analog and digital PET) for predicting para-aortic LNM in cervical cancer. Results showed that the constructed ML model had an extremely high predictive ability, with an AUC of 0.96. Zhang et al. [119] showed that the constructed ML model with the LR algorithm using the PET-radiomics scores established using the LASSO regression had good predictive performance for not only pelvic LNM (AUC: 0.817) but also the expression of COX-2 (AUC: 0.814) in cervical cancers. Li et al. [120] revealed that the ML model with the LR algorithm using the PET-radiomics scores constructed using the LASSO regression had good predictive performance for LVSI in cervical cancer, with an AUC of 0.806. Chong et al. [121] showed that the constructed ML model with the SVM algorithm using conventional parameters (SUVmax, MTV, and TLG) and selected 29 PET-radiomics using the LASSO regression algorithm had good predictive performance for intratumoral budding in cervical cancer (AUC: 0.784).

### Predicting treatment response or survival

A few reports have addressed the efficacy of ^18^F-FDG PET/CT radiomics-based ML analysis for predicting treatment outcomes or prognosis in cervical or endometrial cancer [122–124] (Table 6).

Ferreira et al. [122] showed that the ML model with the RF algorithm using clinical data and PET-radiomics had good performance for predicting disease-free survival in patients with advanced-stage cervical cancer who received CRT (AUC: 0.78). Another study revealed that the ML model with the Naïve Bayes algorithm constructed based on FIGO stage and four pretreatment PET-radiomics features (including surface area, MTV, GLRLM_RLNU, and GLRLM_GLNU) was a significant predictor of PFS (HR:6.89, *p* = 0.003) in patients with cervical cancer who underwent surgery and/or received CRT or chemotherapy [123]. In endometrial cancers, the ML model with the kNN algorithm established using combined clinical data and PET-radiomics has been useful for predicting disease progression, with an AUC of 0.890 [124]. In this study, coarseness, which was the best PET-radiomics feature, was considered a significant and independent factor of PFS (HR:0.65, *p* = 0.003) and OS (HR:0.52,* p* < 0.001) in the multivariate Cox regression analysis.

## Summary

In cervical or endometrial cancers, each best ML model had good predictive performance for predicting tumor stage with an AUC or accuracy of > 0.70. Moreover, the best ML model or best PET-radiomics feature is a significant predictor of survival, and the heterogenous ML approaches were also observed among the reported studies. Although there are not so many reports that have explored the usefulness of ^18^F-FDG PET/CT radiomics-based ML analysis associated with gynecological tumors, the ^18^F-FDG PET/CT radiomics-based ML analysis might provide useful information about patient management with gynecological tumors for clinicians.

## Clinical application of ^18^F-FDG PET/CT radiomics-based ML analyses in other tumors

In hematological malignancies including multiple myeloma and acute leukemia, ^18^F-FDG PET/CT radiomics-based ML analyses have been applied to identify skeletal metastases, predict diffuse infiltration in the bone marrow, or predict prognosis [125–128] (Table 7).

**Table 7:** Summary of representative studies on ^18^F-FDG PET/CT radiomics-based machine learning analyses in other types of tumors

Authors	Years	Tumor type	Aim	Sample size	Constructed ML models	Core ML algorithm	Best ML model	Validation	Results^a^
**Differentiating primary from metastatic tumors**									
Mannam et al. [125]	2022	MM	MM vs. skeletal metastasis	*n* = 40	CT radiomics-based model PET radiomics-based model Combined model	Multilayer perceptron	Combined model	Training and validation cohorts	AUC: 0.9538
**Predicting tumor stage, treatment response, or survival**									
Mesguich et al. [126]	2021	MM	Diffuse infiltration in the bone marrow	*n* = 30	Combined CT + PET radiomics-based model	RF	–	Training and validation cohorts	AUC: 0.90
Li et al. [127]	2019	Acute leukemia	Diffuse infiltration in the bone marrow	*n* = 41	Combined CT + PET radiomics-based model	RF	–	Training and validation cohorts	Accuracy: 0.886
Ni et al. [128]	2023	MM	PFS	*n* = 98	Clinical model Combined PET and CT radiomics-based model Combined clinical, PET radiomics-based, and CT radiomics-based model	LASSO + cox regression	Combined clinical, PET radiomics-based, and CT radiomics-based model	Training and validation cohorts	C-index: 0.698
Feng et al. [130]	2022	Neuroblastoma	MKI status	*n* = 102	Clinical model Combined PET and CT radiomics-based model Combined clinical, PET, and CT radiomics-based model	XGB	Combined PET and CT radiomics-based model	Training and validation cohorts	AUC: 0.951

*AUC* area under the receiver operating characteristic curve, *C-index* concordance index, *LASSO* least absolute shrinkage and selection operator algorithm, *MKI* mitosis-karyorrhexis index, *ML* machine learning, *MM* multiple myeloma, *PFS* progression-free survival, *RF* random forest, *XGB* gradient tree boosting

^a^Performance only presents the result of the best machine learning model

Mannam et al. [125] showed that the ML model with the multilayer perceptron algorithm established based on CT-radiomics and PET-radiomics had good classification accuracy between multiple myeloma and skeletal metastases, with an AUC of 0.9538. Mesguich et al. [126] developed an ML model with the RF algorithm using five PET/CT radiomics for predicting diffuse infiltration in the bone marrow in multiple myeloma. Results showed that the constructed ML model had an extremely high predictive ability, with an AUC of 0.90. Further, the ML model with the RF algorithm using CT-radiomics and PET-radiomics had good performance in predicting bone marrow involvement in acute leukemia [127]. The diagnostic accuracy of this model was significantly higher than that of visual analysis (0.886 vs. 0.686, *p* = 0.041). Ni et al. [128] evaluated the ability of ^18^F-FDG PET/CT radiomics-based ML analysis for predicting PFS after multiple myeloma treatment. Results showed that the ML model with the LASSO + cox regression algorithm trained using the combined clinical and PET/CT radiomics-based model had a higher predictive performance (C-index: 0.698) than the ML model with clinical data (C-index: 0.563) or PET/CT radiomics-based model (C-index: 0.651) alone.

The mistosis-karyorrhexis index (MKI) status is an independent prognostic factor of neuroblastoma [129]. Feng et al. [130] developed the ^18^F-FDG PET/CT radiomics-based ML model for predicting MKI status in neuroblastoma. The constructed ML model with the XGB algorithm using PET/CT radiomics had an extremely high predictive ability, with an AUC of 0.951. Thus, the ML model can be used to noninvasively predict MKI status in pediatric neuroblastoma. Further, it is a significantly effective tool for the long-term management of pediatric neuroblastoma.

## Conclusion

The efficacy of ^18^F-FDG PET/CT radiomics-based ML analyses in various tumors was investigated. The number of studies about this topic has been increasing after 2018. The ^18^F-FDG PET/CT radiomics-based ML analyses might be expected to be important tools for patient management in several types of tumors. However, previous studies have reported numerous ML procedures including the use of algorithms, and different ML models have been applied for the same purpose. Thus, various approaches are used to perform ^18^F-FDG PET/CT radiomics-based ML analysis in oncology. Moreover, ^18^F-FDG PET/CT radiomics-based ML models, which can be easily and universally applied in clinical practice, should be established.
